# Multiplexed fluorescence and scatter detection with single cell resolution using on-chip fiber optics for droplet microfluidic applications

**DOI:** 10.1038/s41378-024-00665-w

**Published:** 2024-03-12

**Authors:** Preksha Gupta, Ambili Mohan, Apurv Mishra, Atindra Nair, Neeladri Chowdhury, Dhanush Balekai, Kavyashree Rai, Anil Prabhakar, Taslimarif Saiyed

**Affiliations:** 1 Discovery Innovation Accelerator, Centre for Cellular and Molecular Platforms, Bellary Road, Bengaluru, 560065 Karnataka India; 2 Department of Electrical Engineering, Indian Institute of Technology Madras, Chennai, 600036 Tamil Nadu India

**Keywords:** Optical sensors, Engineering, Optical physics

## Abstract

Droplet microfluidics has emerged as a critical component of several high-throughput single-cell analysis techniques in biomedical research and diagnostics. Despite significant progress in the development of individual assays, multiparametric optical sensing of droplets and their encapsulated contents has been challenging. The current approaches, most commonly involving microscopy-based high-speed imaging of droplets, are technically complex and require expensive instrumentation, limiting their widespread adoption. To address these limitations, we developed the OptiDrop platform; this platform is a novel optofluidic setup that leverages the principles of flow cytometry. Our platform enables on-chip detection of the scatter and multiple fluorescence signals from the microfluidic droplets and their contents using optical fibers. The highly customizable on-chip optical fiber-based signal detection system enables simplified, miniaturized, low-cost, multiparametric sensing of optical signals with high sensitivity and single-cell resolution within each droplet. To demonstrate the ability of the OptiDrop platform, we conducted a differential expression analysis of the major histocompatibility complex (MHC) protein in response to IFN*γ* stimulation. Our results showed the platform’s ability to sensitively detect cell surface biomarkers using fluorescently labeled antibodies. Thus, the OptiDrop platform combines the versatility of flow cytometry with the power of droplet microfluidics to provide wide-ranging, scalable optical sensing solutions for research and diagnostics.

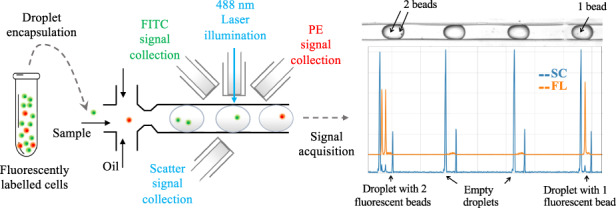

## Introduction

Droplet-based microfluidic technologies provide a variety of solutions for high-throughput single-cell analysis^1,2^ at the genomic^3^, epigenomic^4^, transcriptomic^5–7^, proteomic^8,9^ and metabolomic^10^ levels, with increasing applications in the fields of immunotherapies^11,12^, precision medicine^13^, regenerative medicine^14^, drug development^11,15^ and diagnostics^10,16^. The successful translation of several such droplet-based techniques into commercial assays (e.g., ddPCR by Bio-Rad and Chromium by 10X Genomics) has led to the unprecedented adoption of microfluidic technologies in the commercial sector. The power of droplet microfluidics stems in part from the ability to compartmentalize a larger fluid volume and its contents into much smaller nanoliter-sized water-in-oil emulsion droplets. The resultant droplets act as microreactors for the rapid analysis of encapsulated single cells and can be used for complex multistep biological and chemical assays with considerably decreased reagent consumption and much faster sampling rates. Miniaturization of reaction volumes lowers the overall cost of the assays and enables rapid detection of secreted biomolecules from encapsulated cells as they quickly reach detectable levels within the smaller droplet volume^1,12^. Furthermore, droplets can be flexibly manipulated to split, merge, incorporate new reagents within the preformed droplets, selectively sort out sub-populations of interest and broken down to recover viable cells^1,12,17–19^.

One key component of droplet-based assays is the detection and analysis of droplet contents. Despite the development of increasingly complex applications of droplet-based assays, there has been very limited progress in the sensing of cells or molecules secreted by cells trapped inside droplets. Fluorescence readouts from droplets are the most common approach for assessing droplet contents^20^. Traditional microfluidic setups rely on fluorescence microscopy instrumentation for signal detection and high-speed camera imaging of droplets for validation^12,17,18,21–23^. This off-chip optical setup often involves complex integration and alignment of conventional free-space optics with arrays of lenses and dichroic mirrors. The entire system is rather expensive and bulky, requires skilled handling and maintenance, has limited flexibility for assay design due to fixed optical components, and is restricted in terms of the number of optical parameters that can be simultaneously measured^20,23^. The cost and technical complexity of these optical setups have created a high-entry barrier limiting the adoption of droplet microfluidic technologies for conventional biological research and their applications in point-of-care diagnostics.

In addition to fluorescence microscopy, flow cytometry has been the technology of choice for high-throughput optical analysis of single cells in conventional research and diagnostic setups^24^. In flow cytometry, light scattering and fluorescence emission signals generated by a beam of incident light illuminating a stream of single cells provide unparalleled multiparametric cellular analysis^25^. There have been several attempts to miniaturize and apply the principles of flow cytometry in lab-on-chip devices for the analysis of single cells flowing through an optofluidic channel^26–32^. However, the application of flow cytometry principles for the analysis of single cells encapsulated in water-in-oil droplets remains challenging, in part due to the confounding optics of light passing through phases of different refractive indices coupled with the droplet size often being much larger than the cell within it. A few studies have demonstrated the detection of scatter, absorbance and fluorescence signals from dye solutions and cell populations encapsulated in droplets^12,17,21,33–36^. However, studies thus far lack the sensitivity and the ability to detect and differentiate multiple optical signals with single-cell resolution required for optimal high-throughput analysis. Attempts have also been made to study cells in droplets in a standard flow cytometer by making double emulsion droplets that can be suspended in aqueous sheath fluid^37^. While being a powerful technique, double emulsion droplets are still challenging to work with and cannot be adapted for the versatile droplet-based workflows currently being used^24^.

One approach to simplify and miniaturize the optical detection of droplets is to develop on-chip optofluidics by integrating micro-optical components such as lenses, waveguides and optical fibers within the chip design^29,32,38–40^. In particular, microfluidic chip-integrated optical fibers have been demonstrated to function as effective waveguides for both the illumination of droplets by incident light and the collection of optical signals from droplets^33,35,36^. Owing to their lower cost, ease of integration, compatibility with a wide range of wavelengths, negligible transmission loss and flexibility of use, optical fibers can provide appropriate optical sensing solutions across diverse microfluidic devices.

**Fig. 1:**
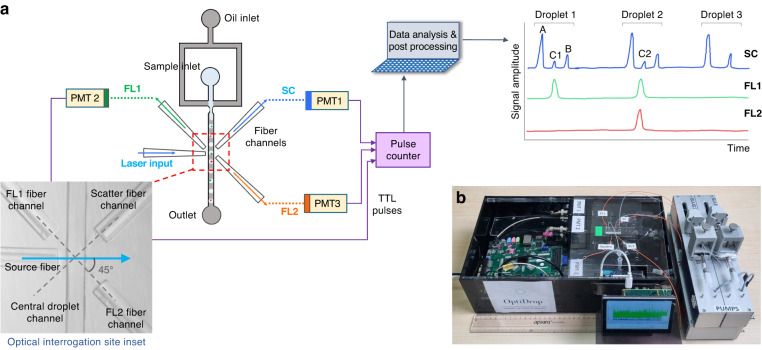
Device setup. **a** The OptiDrop platform comprises a microfluidic chip with an oil and water inlet for the formation of droplets at the flow-focusing junction. The aqueous phase is encapsulated within droplets and subsequently flows through the optical interrogation site (inset) flanked by a set of optical fiber grooves arranged around the central channel. The grooves on the chip are used to house the optical fibers at set angular positions, allowing for effective droplet illumination with the incident laser light and collection of scattered light and fluorescence signals as the droplet passes through the light beam. The optical fiber output is coupled into a PMT for detection. TTL pulse counts from each PMT are integrated by an FPGA chip with a pulse counter and plotted as raw signal intensity peak data. The raw data can be further analyzed to identify or measure fluorescence intensities from the cells of interest. **b** Benchtop assembled unit with a foot ruler for scale along with a live data stream viewing screen and syringe pumps

Here, we demonstrate the integration of optical fibers on a microfluidic chip for multiparametric scatter and fluorescence analysis of droplet contents with single-cell resolution (Fig. 1). The droplet interrogation point on the microfluidic channel is flanked by a set of optical fibers placed at specific angles to the incident light fiber. The novel placement of fibers on the OptiDrop chip enables the collection of light scattered both off the surface of the droplet and from its contents. It also enables highly sensitive detection of multiple fluorescence signals emanating from both the solvent phase (dye/chemical solutions or secreted molecules) and from each cell or bead-associated fluorophore. Thus, by using a single laser as the light source and multiple photomultiplier tubes (PMTs) for signal detection, the corresponding scatter and fluorescence signals from each droplet are recorded as electronic signal peaks. The recorded peak data can be used for real-time visualization and further data analysis. This simultaneous acquisition of scatter and multiplexed fluorescence signals from each droplet combines the flexibility of optofluidics with the versatility of flow cytometry and provides unparalleled optical detection capabilities for droplet-based assays. The use of on-chip optical fibers for light collection avoids the use of any free-space optical components, enabling miniaturization at a much lower cost. The setup can be easily customized per the assay requirement for the combination of laser source and fluorophores by simply changing the set of optical filters attached to the PMTs for detecting the appropriate wavelengths of light.

For validation, we characterized the performance of the OptiDrop platform using standard industry dyes and fluorescent-labeled intensity standard beads. We further demonstrated its biological relevance using one of the most common techniques for detecting cell surface biomarkers via the use of fluorescently tagged antibodies. The expression of major histocompatibility complex (MHC) proteins on the cell surface in response to an immunogen, cytokine or external infection is often used to monitor the activity of an organism’s adaptive immune response^41^. Here, we showed that the OptiDrop platform is capable of simultaneously detecting differences in the cell surface expression of MHC I and MHC II molecules in response to interferon-gamma (IFN*γ*) activation in mouse embryonic fibroblast (MEF) cells encapsulated within droplets. To our knowledge, this represents the first demonstration of multiplexed fluorescence and scatter signal detection with single-cell resolution using on-chip optofluidics.

## Results

### Design and characterization of the OptiDrop platform

The OptiDrop platform comprises a microfluidic chip, optical fibers for laser coupling and optical signal collection, photomultiplier tubes (PMTs) for optical signal detection, and a pulse counter for signal acquisition along with algorithms for signal processing and analysis (Fig. 1). The microfluidic device has a standard flow-focusing junction that allows the generation of stable monodisperse nanoliter-sized water-in-oil droplets over extended periods of time^5,42^. For on-chip optical analysis, the central droplet flow channel has a set of coplanar grooves or fiber guide channels set at an angle of 45° to the incident laser guide channel at the point of interrogation (Fig. 1 inset). Optical fibers placed inside the guide channels are used both for illumination with a 488 nm fiber-coupled laser and for the collection of scattered and fluorescent light signals from the droplet passing through the point of interrogation. Optical signals collected via optical fibers are individually coupled into a photodetector, in this case, a PMT with TTL (transistor-transistor logic) output. As each droplet flows through the incident beam of light, the corresponding PMT outputs are read over a period using a pulse counter. The acquired signals are captured and processed to simultaneously obtain the corresponding scatter and up to three fluorescence signals from each droplet and its encapsulated contents. The resultant on-chip optofluidic setup, along with a live data viewing screen and syringe pumps, can currently be assembled into a compact benchtop unit approximately 20”×12”×4” in dimensions (Fig. 1b). By avoiding the use of free space optical components and microscope-associated video camera unit, we not only miniaturize the setup but also significantly reduce the overall cost of the system. The current setup costs about an INR of 10,00,000 (or USD 12,500) are mostly attributed to the cost of a 100 mW laser, three PMTs and syringe pumps. Depending on the application requirements, the cost of light sources, detectors and fluidic pumps can easily be further reduced by replacing these components with lower-cost alternatives for larger-scale production without compromising the sensitivity of the device. Thus, the OptiDrop platform can enable wider-scale adoption for research and development activities in academic laboratories and industry along with on-site applications in primary health care centers.

### Droplet scatter signal

**Fig. 2:**
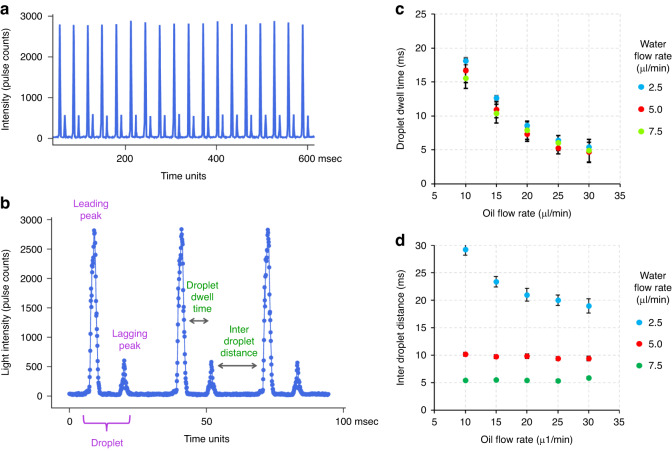
Droplet scatter signal. **a** Raw PMT data output as a count of light pulses coupled into the PMT via an optical fiber over time. **b** As each droplet crosses the incident beam, it results in a characteristic two peak scatter signal of a leading and a lagging peak. Each point on the plot represents the actual pulse count from the PMT. **c** The distance between the leading and lagging peaks of the droplet or the ‘droplet dwell time’ is a function of the oil flow rate and is independent of the aqueous flow rate. **d** The distance between droplets is mainly determined by the aqueous flow rate. The error bars indicate the standard deviation

As each droplet travels through the incident beam, light is scattered off its surface and its contents. Based on Mie scattering theory^43^ and observations made by Mohan et al.^29^, the scattered light is collected via an optical fiber placed at an angle of 45° in the forward direction. The collected scatter signal is coupled into a PMT mounted with a combination of bandpass and neutral density filters. The pulse counter reads the PMT TTL pulses over a specified period and transmits the count value represented as points, as shown in Fig. 2. The amount of light scattered off the droplet surface is a function of the difference in refractive indices of the media as light passes from the oil phase to the aqueous phase^44^. A combination of surface reflection, refraction and total internal reflection at the droplet boundary results in the signature two-peak droplet scatter signal (Fig. 2b). The tall leading peak corresponds to the front of the droplet entering the incident light, and the short lagging peak corresponds to the rear end of the droplet leaving the incident laser light. Thus, the leading and lagging scatter peaks effectively represent the droplet boundaries.

The droplet scatter signals were further characterized by generating droplets at varying oil and water flow rates. An increase in the oil flow rate with a constant aqueous flow rate results in a corresponding reduction in the intra-droplet peak distance. Since the oil flow rate determines the velocity of flowing droplets^45^, faster-flowing droplets spend a correspondingly lesser amount of time crossing the incident beam of light. Thus, the reduced ‘dwell time’ of the droplets results in a reduction in intra-droplet peak distance. Incidentally, the droplet dwell time is independent of the flow rate of the aqueous medium (Fig. 2c). On the other hand, an increase in the water flow rate at a constant oil flow rate results in an increase in the frequency of droplet formation, as evidenced by the reduction in the inter-droplet peak distance (Fig. 2d). Therefore, the oil and aqueous flow rates can be modulated as per requirement to generate and optically analyze droplets at a frequency range of 20–100 Hz while ensuring optimal intra- and inter-droplet peak separations for downstream data analysis. While the electronics can cope with up to 10 times higher droplet frequencies, the current setup is limited by the fluidics of the larger size droplets.

**Fig. 3:**
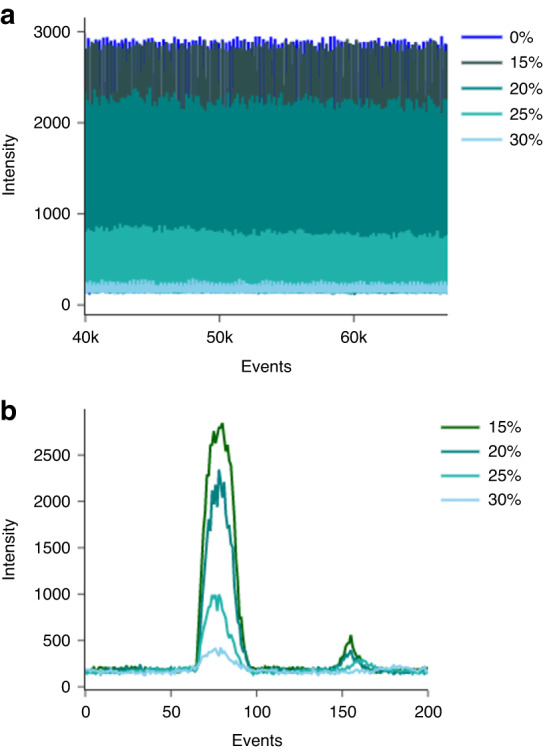
Impact of 3-bromobenzotrifluoride addition on the droplet scatter signal. Increasing concentrations of 3-bromobenzotrifluoride (0-30%) added to Novec dSURF oil reduce the refractive index difference between the oil and aqueous phase, leading to a corresponding reduction in the intensity of the scatter signal as more light passes through the droplet without being refracted off its surface into the scatter collection fiber. **a** Scatter signal from multiple droplets. **b** Scatter signal from single droplet

To maximize the amount of incident light entering the droplet and the emitted light collected by the optical fibers, the refractive index of the oil was modified to match that of the aqueous phase^46^ by adding 3-bromobenzotrifluoride to the Novec dSURF oil. By varying the concentration of 3-bromobenzotrifluoride, we were able to carefully control the strength of the droplet scatter signal per the assay requirements (Fig. 3).

### Droplet fluorescence signal

**Fig. 4:**
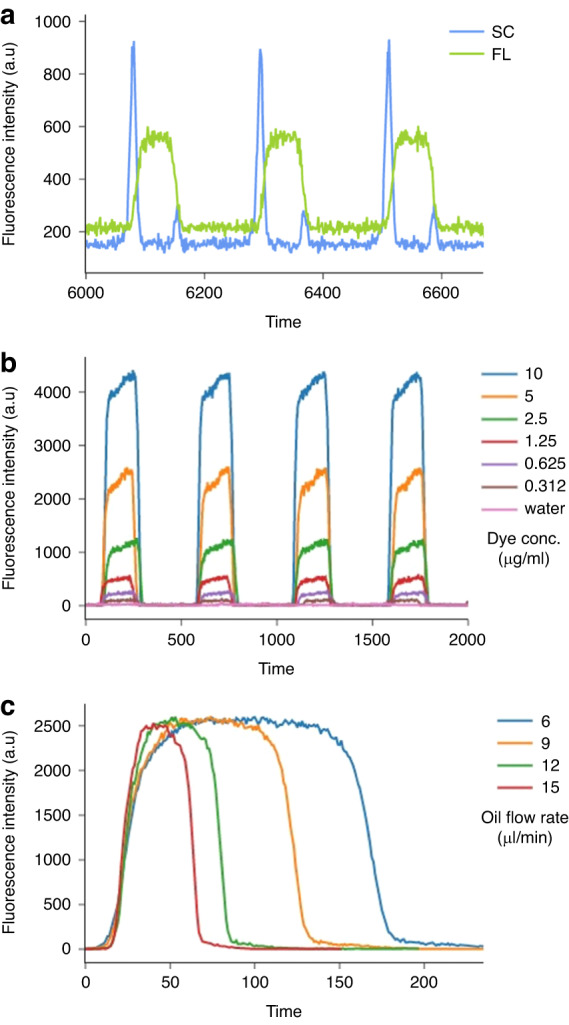
Fluorescence signal characterization of dye droplets. **a** Typical droplet scatter (SC) and fluorescence (FL) signal acquisition from the droplets encapsulating fluorescent dye solutions. **b** Overlap plot of the droplet fluorescence signals from the droplets encapsulating increasing concentrations of Rhodamine 123 dye (0–10 µg/ml) solutions recorded at the same flow rates. **c** Overlap plot of the droplet fluorescence signals acquired from droplets encapsulating the same Rhodamine 123 dye solution at various oil flow rates

High-sensitivity fluorescence readouts are crucial for several chemical and biological assays. The OptiDrop platform can be used to collect as many as four fluorescence signals emitted from a single droplet using optical fibers placed at 45° angles to the laser guide channel (Fig. 1). The light collected through each optical fiber is coupled into a separate PMT with a bandpass filter attached for the corresponding color signal to be recorded. To characterize the fluorescence sensing capabilities of the OptiDrop platform, we first generated dye droplets by using increasing concentrations of the Rhodamine 123 dye as the aqueous phase and measured the fluorescence intensities for each sample (Fig. 4a). At a fixed oil and aqueous flow rate, uniformly formed droplets had a fluorescence signal with a constant peak width and increasing peak height corresponding to the increase in dye concentration in different samples (Fig. 4b). At a fixed dye concentration, the droplet fluorescence peak height remained constant, while the peak width increased with increasing droplet dwell time at slower oil flow rates (Fig. 4c).

**Fig. 5:**
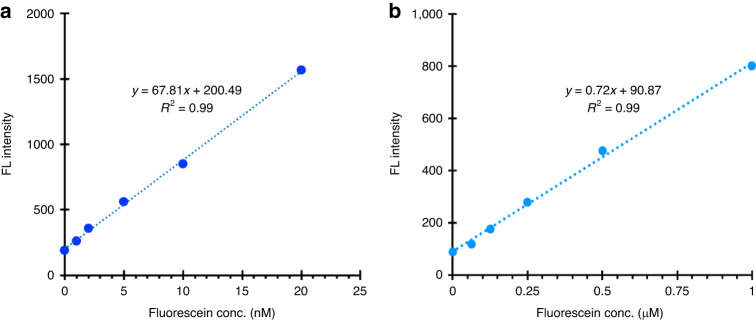
Detection efficiency of fluorescein dye. The fluorescence signal intensity linearly increased with increasing fluorescein dye concentration and exhibited a high dynamic range of **a** 0-20 nM **b** 0-1 µM. The LOD for fluorescein was determined to be 1 nM

We then examined the linearity and dynamic range of detection of the OptiDrop platform. Increasing dye concentrations of fluorescein resulted in a linear increase (R^2^ value of 0.99) in the droplet fluorescence intensities, with a high dynamic range of detection spanning from 1 nM to 1 µM for 4 nL droplets (Fig. 5). To determine the detection efficiency or sensitivity of the OptiDrop platform, we measured the limit of detection (LOD) for fluorescein (an industry reference standard) and phycoerythrin dye droplets. The LOD, defined as the concentration that produces a signal equal to three times the standard deviation of the blank measurements, was determined to be 1 nM fluorescein (Fig. 5). Similarly, the LOD for phycoerythrin was found to be as low as 0.5 pM (Fig. S1^†^). Fluorescence signals were also measured on a standard benchtop fluorimeter (VarioScan lux, Thermo Fisher Scientific) by loading 20 *µ*L of the dye solutions in a 384-well plate for comparison (Fig. S2^†^). The benchtop fluorimeter required a much larger sample volume (20 μL/sample as opposed to 4 nL/droplet) for the 1 nM limit of detection and displayed a much larger margin of error, as indicated by error bars representing the standard deviation of the linear equation, with an R^2^ value of 0.97. These results demonstrate the ability of the OptiDrop platform to detect multiple fluorescence signals within droplets with LODs comparable to or better than those reported previously in similar optofluidic devices with good sensitivity and robustness^35,36^. Here, we have shown representative data for droplets encapsulating single dye solutions, but the setup can further easily be used to detect fluorescence signals from a mixture of dyes encapsulated within droplets by simultaneously collecting optical signals at different angles.

### Encapsulated single-cell/bead scatter and fluorescence signals

**Fig. 6:**
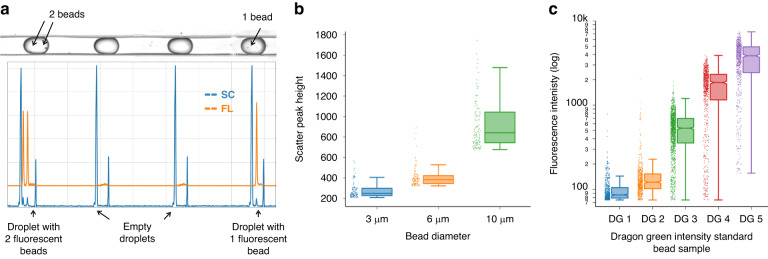
Scatter and fluorescence signals from the beads encapsulated within droplets. **a** Snapshot of the droplets flowing through the microfluidic channel (top image) and the corresponding data recorded on the OptiDrop platform (below plot). Four droplet boundaries are demarcated by the leading and lagging scatter peaks. The bead scatter and fluorescence peak are located within the boundary peaks. The scatter (SC) signal is shown in blue, and the fluorescence (FL) signal is shown in orange. **b** Box plot indicating the size distribution of the scatter signals from 3, 6 and 10 µm sized beads. **c** Box plot demonstrating the increasing fluorescence intensity profiles of the five populations of the Dragon Green intensity standard kit

The OptiDrop platform provides not only whole-droplet scatter and fluorescence signals but also multiparametric optical information from each droplet constituent cell/bead with single-cell resolution. During a typical run, the scatter and corresponding fluorescence signals from each cell/bead are captured as individual peaks within the droplet boundary scatter peaks (Fig. 6a). For instance, in a sample of fluorescent microbeads randomly distributed in droplets following a Poisson distribution, the OptiDrop platform can be used to effectively distinguish between droplets containing zero, one or two beads. A double bead-containing droplet has 2 peaks each corresponding to the bead scatter and fluorescence signals present within the leading and lagging droplet scatter peaks (Fig. 6a). The height of the scatter peaks roughly corresponds to the bead size. An increase in the size (3, 6 and 10 µm) of the polystyrene particles encapsulated in droplets resulted in a corresponding increase in their scatter signal intensity (Fig. 6b). Similarly, the height of the fluorescence peaks corresponds to the signal intensity associated with each cell/bead. To characterize the platform’s ability to measure relative fluorescence intensities, we used the Dragon Green intensity standard kit (Bangs Laboratories, Inc.). The OptiDrop platform can effectively distinguish between the five populations of polystyrene microbeads dyed with increasing amounts of Dragon Green fluorophore (Fig. 6c).

To determine the limit of detection for bead- or cell-associated fluorescence, we used Rainbow flow cytometry calibration particles (Spherotech) as the recommended standards for biological applications. The peak 2 bead population corresponds to the dim beads often used in standard flow cytometers to set the instrument for detecting the lowest fluorescence signal measured above the blank signal, while the peak 5 population corresponds to the brighter end of the spectrum of bead fluorescence. Fluorescent signals from both the dim peak 2 and the bright peak 5 population of beads were effectively captured (Fig. S3^†^).

**Fig. 7:**
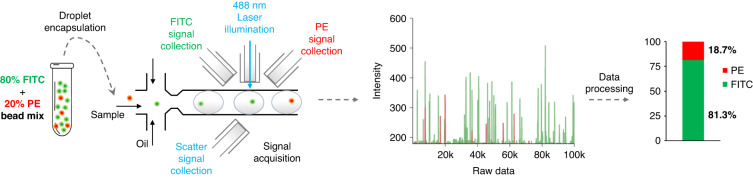
Effective signal acquisition and differentiation in a mixed bead population. A mixture of FITC (80%) and PE (20%) stained CaliBRITE bead sample data is analyzed on the OptiDrop platform. The acquired raw fluorescence peak signals are analyzed, and the proportions of FITC and PE beads are calculated, as shown in the bar plot

To validate the robustness of the OptiDrop platform, 80% FITC-stained CaliBRITE beads were mixed with 20% PE-stained CaliBRITE beads. FITC and PE fluorescent signals were acquired from the mixed bead sample encapsulated within droplets and on a standard flow cytometer for comparison. As shown in Fig. 7, the distribution of FITC and PE beads on the scatter plot recorded with the OptiDrop platform revealed the expected 8:2 signal ratio. Similar results were observed via standard flow cytometry (Fig. S4^†^). This experiment demonstrated the platform’s ability to effectively capture fluorescence readouts from each bead in the population.

### Biological validation: MHC cell surface expression profiling in MEFs

Antibody-based protein biomarker detection is the most common method for assessing the expression of cell surface proteins via flow cytometry and has versatile applications in both biomedical research and diagnostics. One such application is the analysis of MHC immunomodulation in cells. MHC proteins play a critical role in the immune system by presenting antigens to T cells. MHC molecules are divided into two main classes: MHC class I and MHC class II molecules. MHC class I molecules are expressed on the surface of most nucleated cells and present antigenic peptides derived from intracellular pathogens, such as viruses or intracellular bacteria, to CD8+ cytotoxic T cells. On the other hand, MHC class II molecules are expressed primarily on the surface of antigen-presenting cells, such as dendritic cells, macrophages, and B cells. These molecules present antigenic peptides derived from extracellular pathogens, such as bacteria or parasites, to CD4+ helper T cells. Together, the MHC class I and II molecules enable the detection and elimination of infected cells by activating the appropriate immune response^47^. Thus, the expression levels of MHC molecules on the surface of cells can serve as an important indicator of an organism’s ability to fight pathogens. Recent studies have suggested that specific MHC alleles and their expression may be associated with increased susceptibility to COVID-19 infection or more severe disease outcomes^48,49^. Additionally, abnormalities in MHC expression can be used as a diagnostic tool for various diseases, including autoimmune disorders and cancer^50,51^.

**Fig. 8:**
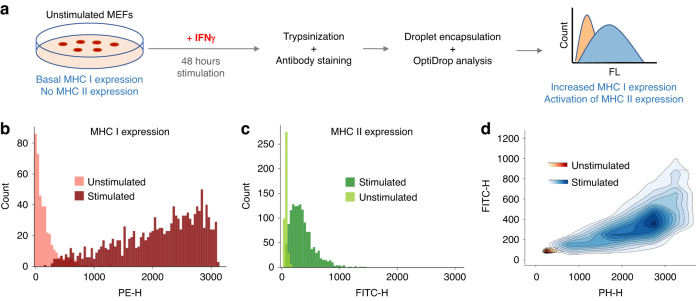
MHC protein expression profiling in response to IFN*γ* stimulation. **a** Schematic of the biological assay depicting the stimulation of MEFs with IFN*γ* for 48 hours, subsequent removal of cells from the culture plate using trypsin and staining with PE-tagged MHC I and FITC-tagged MHC II antibodies. The stained cells were encapsulated within droplets on the OptiDrop platform, and the fluorescence signal was acquired. **b**, **c** Fluorescence intensities of each cell from the stimulated and unstimulated MEF samples plotted as histograms depicting MHC I and MHC II expression. **d** Two-color fluorescence density contour plot depicting the relative increase in both MHC I and MHC II expression upon stimulation with IFN*γ*

Given the importance of MHC proteins in understanding immune system function and developing therapeutic strategies for immune-related diseases, we analyzed the expression of these proteins in mouse embryonic fibroblasts (MEFs) in response to IFN*γ* activation. Utilizing the ability of the OptiDrop platform to simultaneously detect scatter and two fluorescence signals from each encapsulated cell, we used PE- and FITC-conjugated antibodies to stain cell surface MHC class I and class II proteins, respectively. The stained cells were encapsulated in droplets, and multiparametric analysis was performed on the OptiDrop platform. Unstimulated MEFs have low basal expression of MHC class I proteins and do not express any MHC class II molecules. The OptiDrop platform was effectively able to detect the increase in MHC class I proteins and the activation of MHC class II expression in response to IFN*γ* stimulation (Fig. 8). The results obtained from the OptiDrop platform were validated and found to be comparable to those obtained on a standard flow cytometer (Fig. S5^†^). Both OptiDrop and flow cytometry data indicated that 94% of MHC class I-expressing cells also expressed MHC class II proteins upon IFN*γ* stimulation.

## Discussion

We developed a novel platform for simultaneous scatter and multiplexed fluorescence signal detection from solutions and cells/beads encapsulated within microfluidic droplets with single-cell resolution. The OptiDrop platform is sensitive (1 nM fluorescence detection limit), has a high dynamic range of detection for both solution- and cell/bead-based fluorescence, and can differentiate cells/beads of different sizes based on their scatter signal. To the best of our knowledge, this platform is the first nonimaging platform capable of integrating the principles of flow cytometry for high-throughput multiparametric single-cell analysis of microfluidic droplets.

The OptiDrop platform has several advantages over the existing droplet optical analysis platforms. First, the simplified on-chip fiber optics overcome the limitations of cost, complexity, bulkiness, and lack of flexibility associated with traditional methods of fluorescence microscopy-based high-speed video imaging. The replacement of expensive, bulkier free-space optical components with multimode optical fibers enables miniaturization, significantly reduces cost, and circumvents the need for complex alignments and maintenance while making it easily customizable based on assay requirements. Moreover, the imaging of high-throughput droplet-based assays results in large datasets requiring expensive storage and processing solutions, further limiting their scalability. The live data visualization, small data storage footprint and simpler data analysis algorithm incorporated within the OptiDrop platform further enhances its applicability as a benchtop clinical technology. The features and capabilities of the OptiDrop platform are further compared with those of previously reported droplet optical analysis studies and are presented in Table S1^†^.

Similarly, while the current commercial flow cytometers are highly proficient and versatile in biomedical applications, their applicability is limited in regard to analyzing cells encapsulated within water-in-oil droplets. Additionally, flow cytometers are often used only for endpoint analysis, precluding any dynamic monitoring of single-cell responses over time. The most important distinction of flow cytometers from the OptiDrop platform stems from the ability of microfluidic droplets to act as microchambers for single cells or organoids and their extracellular secretions. Droplet-based assays, and thus in essence, the OptiDrop platform, can not only provide information from single cells but can also detect secreted molecules encapsulated within each respective droplet. Another critical feature of the OptiDrop platform is its ‘closed’ nature; the biological sample remains within easily replaceable tubing and microfluidic cartridges, and never passes through a system open to external contamination. This closed optical flow detection system, as opposed to the open system of standard and imaging flow cytometers such as ImageStream, is particularly suited for clinical applications.

Although we validated the effectiveness of the OptiDrop platform for profiling cell surface protein expression using antibodies, its simplicity allows seamless integration with the multiverse of existing droplet microfluidic workflows for optical sensing. Current single-cell genomic technologies provide a high-resolution view of gene expression and other genomic features but fail to capture other aspects of cellular behavior, such as protein expression or cell morphology. Integrating single-cell genomic data with cellular phenotype information obtained by concurrent multiparametric on-chip optical analysis using the OptiDrop platform can provide a more comprehensive understanding of cellular behavior. The reduced cost and complexity of optical analysis can further help to increase the adoption of droplet microfluidics for point-of-care diagnostics.

The current work provides the foundation for further improvements in the speed, sensitivity, accuracy, and cost of optical sensing of single cells encapsulated in droplets. We hope that the range of applications of the OptiDrop platform will be further extended and that it will enable the development of affordable, scalable benchtop droplet microfluidic solutions in biomedical research and diagnostics.

## Materials and methods

### Device fabrication protocol

The microfluidic chips were fabricated using polydimethylsiloxane (PDMS) by standard soft-lithography techniques as previously described^29^. Briefly, the design mold was fabricated on a silicon wafer by UV exposure through a photolithography mask and subsequent development. A curing agent was added to the PDMS base (Sylgard 184 silicone elastomer kit; Dow Corning Corporation) to a final concentration of 10% by weight; the sample was mixed and poured over the mold to a depth of 7 mm. Following degassing for several minutes and cross-linking at 70 °C for 2.5 hours, the polymerized PDMS was peeled off the mold and cut into individual chips. The inlet and outlet ports were punched in the PDMS with a 0.9 mm diameter biopsy punch for the fluidic connections.

The PDMS layer was subjected to an oxygen plasma treatment (Harrick plasma cleaner) with the patterned side facing up and the substrate layer (cleaned glass slides) adjacent to it. A vacuum was created in the plasma chamber for 45 seconds. Oxygen was slowly purged into the plasma chamber until the color of the chamber became dark with a pink periphery. The oxygen purge was stopped upon the color change, and the chamber was left undisturbed for one or two minutes before the vacuum was released to open the chamber. After removal from the chamber, the PDMS device was pressed against a glass slide without excessive pressure for permanent bonding to the glass slide. The bonded device was further placed in a vacuum oven at 70 °C for 25 to 30 minutes. The central fluidic channels were treated with Aquapel^5^ and kept in a hot air oven at 70 °C for 45 minutes before use.

### Device setup

A sample containing any cells, beads or dye solutions comprised the aqueous phase. Fluigent dSURF oil (2% surfactant in 3 M™ Novec™ 7500 fluorinated oil) was used as the continuous phase. The sample and oil inlets were connected to the syringe pumps (NE 300, New Era Pump Systems, Inc.) via polyethylene tubing (BTPE-50, Instech Laboratories, Inc.) for fluid injection. The flow-focusing junction on the microfluidic chip breaks the flow of the aqueous sample to form water-in-oil droplets as the aqueous sample becomes encapsulated within the droplets. The frequency, speed and size of droplets can be controlled by modulating the oil and aqueous sample flow rates in the syringe pump.

Downstream of the flow-focusing junction, the microfluidic chip has grooves on both sides of the central flow channel for housing optical fibers (Fig. 1). Lensed optical fibers (multimode 105 µm core/125 µm cladding with a working distance of 150 µm from Lase Optics Corporation) or nonlensed fibers (standard multimode OM1 fibers with 62.5 µm core/125 µm cladding diameter) were used as excitation fibers for laser light coupling. Nonlensed OM1 fibers were used for collecting scattered light and fluorescent signals from the droplets. The non-lensed optical fibers were stripped off their outer protective jacket layer with only the core and cladding layer remaining. The stripped fibers were cleaved with a fiber cleaver to obtain a flat surface at the fiber tip end. The fiber guide channels/grooves were filled with index-matching fluid (Eagle Photonics). The optical fibers were then manually inserted into the guide channels with careful observation under a stereo microscope ensuring the absence of any air bubbles between the fiber tip and the end of the fiber guide channel. The chip was placed on a holder, and the screws were tightened to secure the fibers in place. The light signals collected from optical fibers were coupled into PMT detectors (Hamamatsu H10682, Sens-Tech P25PC). Neutral density and bandpass filters were placed between the fiber and PMT interface according to the wavelengths of interest and the laser intensity.

### Data collection and signal processing

TTL-level voltage pulses from the PMTs were processed by implementing a pulse counter for each of them using the FPGA (Field Programmable Gate Array) chip on the Digilent Zedboard (Xilinx Zynq 7000 SoC). Verilog and the Xilinx Vivado suite were used to program the hardware on the FPGA to count the pulses and store them in memory (BRAM). The integration time for pulse counting was set to 100 *µ*s within the hardware. The counts from each PMT were read simultaneously from the BRAM using interrupts, then combined into a single ethernet frame and sent out in a continuous data stream using C and XIlinx SDK libraries on the ARM chip. A Python and Qt based GUI was created to stream the live data output and to store the data. The PMT outputs were overlaid on the GUI to obtain the corresponding scatter and fluorescence values at each timestamp. The PMT pulse count data were processed to identify peaks using our custom Python-based signal extraction algorithm. Leading and lagging peaks in the scatter signals were used to mark the droplet boundaries. Within each droplet boundary, individual peaks for cell/bead scatter and fluorescence were identified and assigned to the respective droplet. The data for each peak were analyzed to obtain the corresponding peak height (signal intensity) values.

### Chemicals and reagents

Fluorescein (sodium salt, SRL 55091) and R-phycoerythrin (52412 Sigma Aldrich) were used for making dilutions to determine the lower limit of detection for the setup. 3-bromobenzotrifluoride (Sigma B59004) was mixed with dSURF oil for the refractive index modifications as per the procedure requirements.

Dragon Green intensity standard kit (DG06M) was purchased from Bangs Laboratories, Inc. Rainbow calibration particles (RCP-60-5-2, RCP-60-5-5) were obtained from Spherotech. FITC and PE stained beads were obtained from the three-color BD CaliBRITE Kit (BD Biosciences 340486). Beckman Coulter CytoFLEX Daily QC Fluorospheres (B53230), Spherotech SPHERO Rainbow Calibration Particles, Peak 2 (RCP-60-50-2) and polystyrene DVB-COOH microspheres from Bangs Laboratories, Inc. (PC07001) were used as reference standards for 3, 6, and 10 µm sized beads, respectively.

### Cells and culture conditions

C57BL/6 embryos were eviscerated at day 12.5-13.5, and embryonic fibroblasts were isolated at the Stem Cell Core (InStem, Bangalore). The cells were cultured in DMEM (Dulbecco’s Modified Eagle Medium, Gibco) supplemented with 10% FBS at 37 °C with 5% carbon dioxide. The cells were grown to 70-80% confluence and then treated for 48 hours with 1 µM recombinant mouse interferon-gamma (Sino Biological, China). The treated cells were detached by trypsinization and stained with antibodies against the MHC cell surface proteins.

Briefly, 1×10^6^ cells were transferred into 1.5 mL microcentrifuge tubes (Eppendorf, Germany) and washed twice with ice-cold FACS buffer containing 5% FBS in PBS. The cells were centrifuged at 1000 rpm for 5 minutes in a MiniSpin Plus microcentrifuge (Eppendorf, Germany). The washed cells were resuspended in 150 *µ*L of FACS buffer and treated with an antimouse MHC class I antibody conjugated to PE (clone 28-148) (Invitrogen, USA) at a concentration of 2 *µ*g per sample and an antimouse MHC class II antibody conjugated to FITC (clone M5/114.15.2) (Invitrogen, USA) at a concentration of 5 µg per sample. The cells were stained for 1 hour at a temperature of 4 °C with shaking at 600 rpm in a Thermomixer Comfort (Eppendorf, Germany). The stained cells were washed twice with ice-cold FACS buffer before being resuspended in FACS buffer for analysis. 15% OptiPrep (D1556 Sigma Aldrich) was added to the aqueous sample to prevent settling of cells within droplets.

### Supplementary information

Supplementary data

## References

[1] Matuła K, Rivello F, Huck WTS (2020). Single-Cell Analysis Using Droplet Microfluidics. Adv. Biosys.

[2] Rakszewska A, Tel J, Chokkalingam V, Huck WTS (2014). One drop at a time: toward droplet microfluidics as a versatile tool for single-cell analysis. NPG Asia. Materials.

[3] Pellegrino M (2018). High-throughput single-cell DNA sequencing of acute myeloid leukemia tumors with droplet microfluidics. Genome Res.

[4] Xu Y, Doonan SR, Ordog T, Bailey RC (2020). Translational opportunities for microfluidic technologies to enable precision epigenomics. Anal. Chem..

[5] Macosko EZ (2015). Highly parallel genome-wide expression profiling of individual cells using nanoliter droplets. Cell.

[6] Zheng GXY (2017). Massively parallel digital transcriptional profiling of single cells. Nat. Commun..

[7] Zilionis R (2017). Single-cell barcoding and sequencing using droplet microfluidics. Nat. Protoc..

[8] Granja JM (2019). Single-cell multiomic analysis identifies regulatory programs in mixed-phenotype acute leukemia. Nat. Biotechnol..

[9] Shahi P, Kim SC, Haliburton JR, Gartner ZJ, Abate AR (2017). Abseq: Ultrahigh-throughput single cell protein profiling with droplet microfluidic barcoding. Sci. Rep..

[10] Del Ben F (2016). A Method for Detecting Circulating Tumor Cells Based on the Measurement of Single-Cell Metabolism in Droplet-Based Microfluidics. Angew. Chem. Int Ed. Engl..

[11] Eyer K (2017). Single-cell deep phenotyping of IgG-secreting cells for high-resolution immune monitoring. Nat. Biotechnol..

[12] Mazutis L (2013). Single-cell analysis and sorting using droplet-based microfluidics. Nat. Protoc..

[13] Ayuso JM, Virumbrales-Muñoz M, Lang JM, Beebe DJ (2022). A role for microfluidic systems in precision medicine. Nat. Commun..

[14] Nazari H (2021). Microfluidic-Based Droplets for Advanced Regenerative Medicine: Current Challenges and Future Trends. Biosens. (Basel).

[15] Wang Y (2020). Advances of droplet-based microfluidics in drug discovery. Expert Opin. Drug Discov..

[16] Barbosa AI, Reis NM (2017). A critical insight into the development pipeline of microfluidic immunoassay devices for the sensitive quantitation of protein biomarkers at the point of care. Analyst.

[17] Baret J-C (2009). Fluorescence-activated droplet sorting (FADS): efficient microfluidic cell sorting based on enzymatic activity. Lab Chip.

[18] Caen O (2018). High-throughput multiplexed fluorescence-activated droplet sorting. Microsyst. Nanoeng..

[19] Sciambi A, Abate AR (2015). Accurate microfluidic sorting of droplets at 30 kHz. Lab Chip.

[20] Măriuţa D (2020). Miniaturization of fluorescence sensing in optofluidic devices-a-chip. Microfluid Nanofluid..

[21] Lu H (2017). High throughput single cell counting in droplet-based microfluidics. Sci. Rep..

[22] Qiao Y (2017). Fluorescence-activated droplet sorting of lipolytic microorganisms using a compact optical system. Lab Chip.

[23] Panwar J, Autour A, Merten CA (2023). Design and construction of a microfluidics workstation for high-throughput multi-wavelength fluorescence and transmittance activated droplet analysis and sorting. Nat. Protoc..

[24] Li M, Liu H, Zhuang S, Goda K (2021). Droplet flow cytometry for single-cell analysis. RSC Adv..

[25] Shapiro, H. M. Practical Flow Cytometry. (2003).

[26] Barat D, Spencer D, Benazzi G, Mowlem C, Morgan H (2012). Simultaneous high speed optical and impedance analysis of single particles with a microfluidic cytometer. Lab Chip.

[27] Wu TH (2012). Pulsed laser triggered high speed microfluidic fluorescence activated cell sorter. Lab Chip.

[28] Cho SH, Chen CH, Tsai FS, Godin JM, Lo YH (2010). Human mammalian cell sorting using a highly integrated micro-fabricated fluorescence-activated cell sorter (μFACS). Lab Chip.

[29] Mohan A, Gupta P, Nair AP, Prabhakar A, Saiyed T (2020). A microfluidic flow analyzer with integrated lensed optical fibers. Biomicrofluidics.

[30] Rosenauer M, Buchegger W, Finoulst I, Verhaert P, Vellekoop M (2011). Miniaturized flow cytometer with 3D hydrodynamic particle focusing and integrated optical elements applying silicon photodiodes. Microfluid Nanofluid..

[31] Zhang Y (2016). Optofluidic device based microflow cytometers for particle/cell detection: A review. Micromachines.

[32] Zhao Y, Li Q, Hu X, Lo Y (2016). Microfluidic cytometers with integrated on-chip optical systems for red blood cell and platelet counting. Biomicrofluidics.

[33] Cole RH, Gartner ZJ, Abate AR (2016). Multicolor Fluorescence Detection for Droplet Microfluidics Using Optical Fibers. J. Vis. Exp..

[34] Gaikwad R, Sen AK (2021). An optomicrofluidic device for the detection and isolation of drop-encapsulated target cells in single-cell format. Analyst.

[35] Guo F (2012). A droplet-based optofluidic device for high-throughput quantitative bioanalysis. Anal. Chem..

[36] Hengoju S (2020). Optofluidic detection setup for multi-parametric analysis of microbiological samples in droplets. Biomicrofluidics.

[37] Brower KK (2020). Double emulsion flow cytometry with high-throughput single droplet isolation and nucleic acid recovery. Lab Chip.

[38] Shivhare PK, Prabhakar A, Sen AK (2017). Optofluidics based lab-on-chip device for in situ measurement of mean droplet size and droplet size distribution of an emulsion. J. Micromech. Microeng..

[39] Yang H, Gijs MAM (2018). Micro-optics for microfluidic analytical applications. Chem. Soc. Rev..

[40] Yang T, Stavrakis S, DeMello A (2017). A High-Sensitivity, Integrated Absorbance and Fluorescence Detection Scheme for Probing Picoliter-Volume Droplets in Segmented Flows. Anal. Chem..

[41] Wieczorek M (2017). Major histocompatibility complex (MHC) class I and MHC class II proteins: Conformational plasticity in antigen presentation. Front Immunol..

[42] Lagus, T. P. & Edd, J. F. High throughput single-cell and multiple-cell micro-encapsulation. *J. Vis. Exp.* **64**, 4096 (2012). 10.3791/4096 PMC3471304 22733254

[43] Mie G (1908). Beiträge zur Optik trüber Medien, speziell kolloidaler Metallösungen. Ann. Phys..

[44] Han X, Shen J, Yin P, Hu S, Bi D (2014). Influences of refractive index on forward light scattering. Opt. Commun..

[45] Ibrahim AM, Padovani JI, Howe RT, Anis YH (2021). Modeling of droplet generation in a microfluidic flow-focusing junction for droplet size control. Micromachines (Basel).

[46] Salmon AR (2016). Monitoring Early-Stage Nanoparticle Assembly in Microdroplets by Optical Spectroscopy and SERS. Small.

[47] Kindt, T. J., Goldsby, R. A., Osborne, B. A. & Kuby, J. Kuby Immunology (W. H. Freeman). (2007).

[48] Castelli EC (2021). MHC Variants Associated With Symptomatic Versus Asymptomatic SARS-CoV-2 Infection in Highly Exposed Individuals. Front Immunol..

[49] Yoo JS (2021). SARS-CoV-2 inhibits induction of the MHC class I pathway by targeting the STAT1-IRF1-NLRC5 axis. Nat. Commun..

[50] Choy EH, Kavanaugh AF, Jones SA (2013). The problem of choice: current biologic agents and future prospects in RA. Nat. Rev. Rheumatol..

[51] Slingluff CL (2011). The present and future of peptide vaccines for cancer: single or multiple, long or short, alone or in combination?. Cancer J..

