# Supplementary material for: Multiplexed fluorescence and scatter detection with single cell resolution using on-chip fiber optics for droplet microfluidic applications
Source: Microsyst Nanoeng. 2024 Mar 12;10:35. doi: 10.1038/s41378-024-00665-w (PMC10933342; doi:10.1038/s41378-024-00665-w)

**Supplementary figures:**


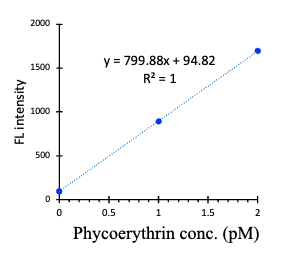


Figure S1: Standard curve for increasing concentrations of Phycoerythrin dye in droplets results in linear increase in fluorescence intensity with an LOD of 1 pM


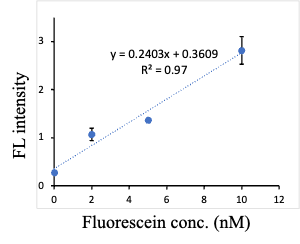


Figure S2: Fluorescence intensity measurement of 20ul Fluorescein dye solutions of increasing concentrations on a standard fluorimeter using a 384 well microplate – Varioscan LUX from Thermo Fisher. LOD was observed to be 2nM. Larger margin of eror as indicated by error bars depicting standard deviation on the linear equation with a R^2^ value of 0.97.


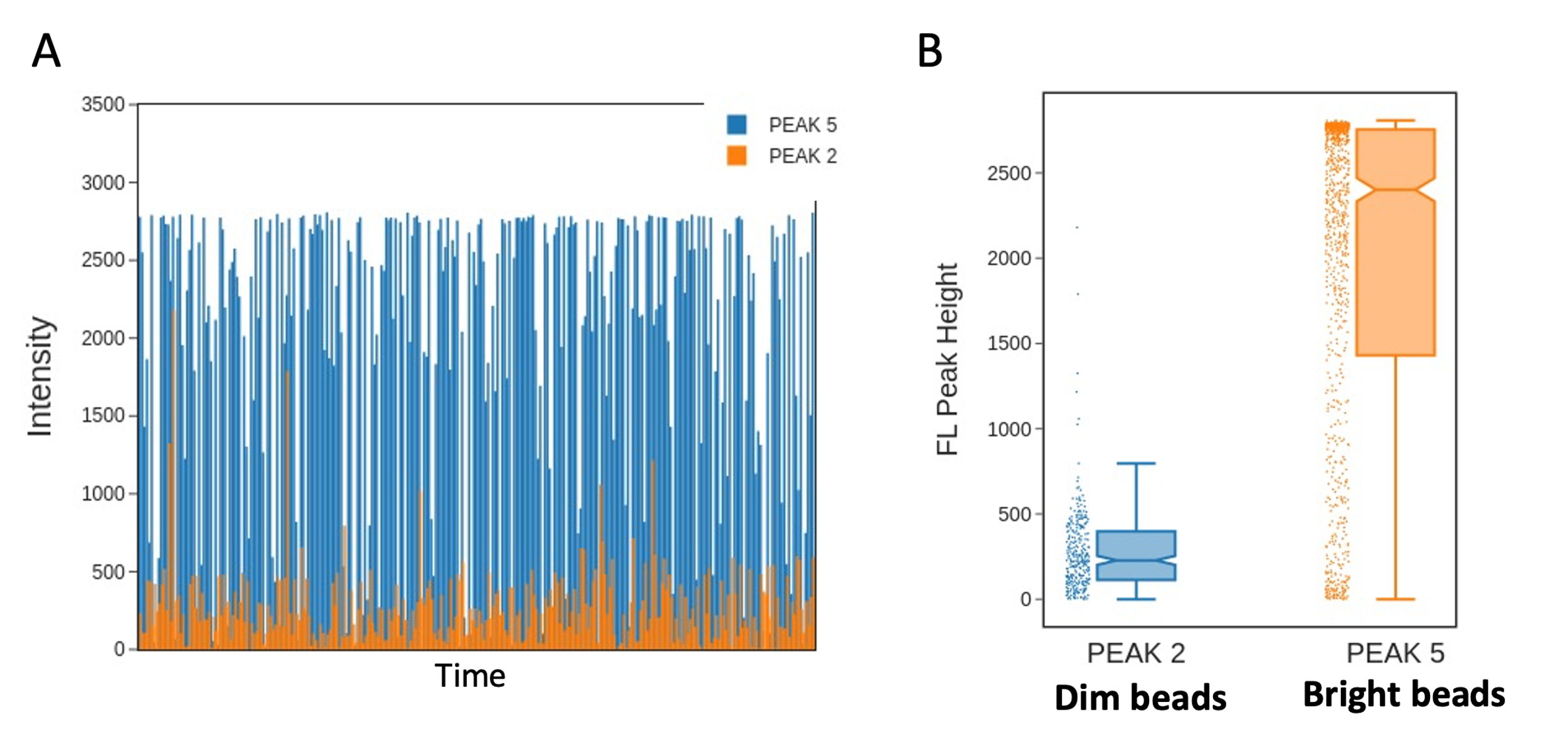


Figure S3: Fluorescence intensity measurements from the Peak 2 (dim) and Peak 5 (bright) populations of the Rainbow flow cytometry calibration particles (Spherotech) A) Raw data B) Box plot of fluorescence peak heights


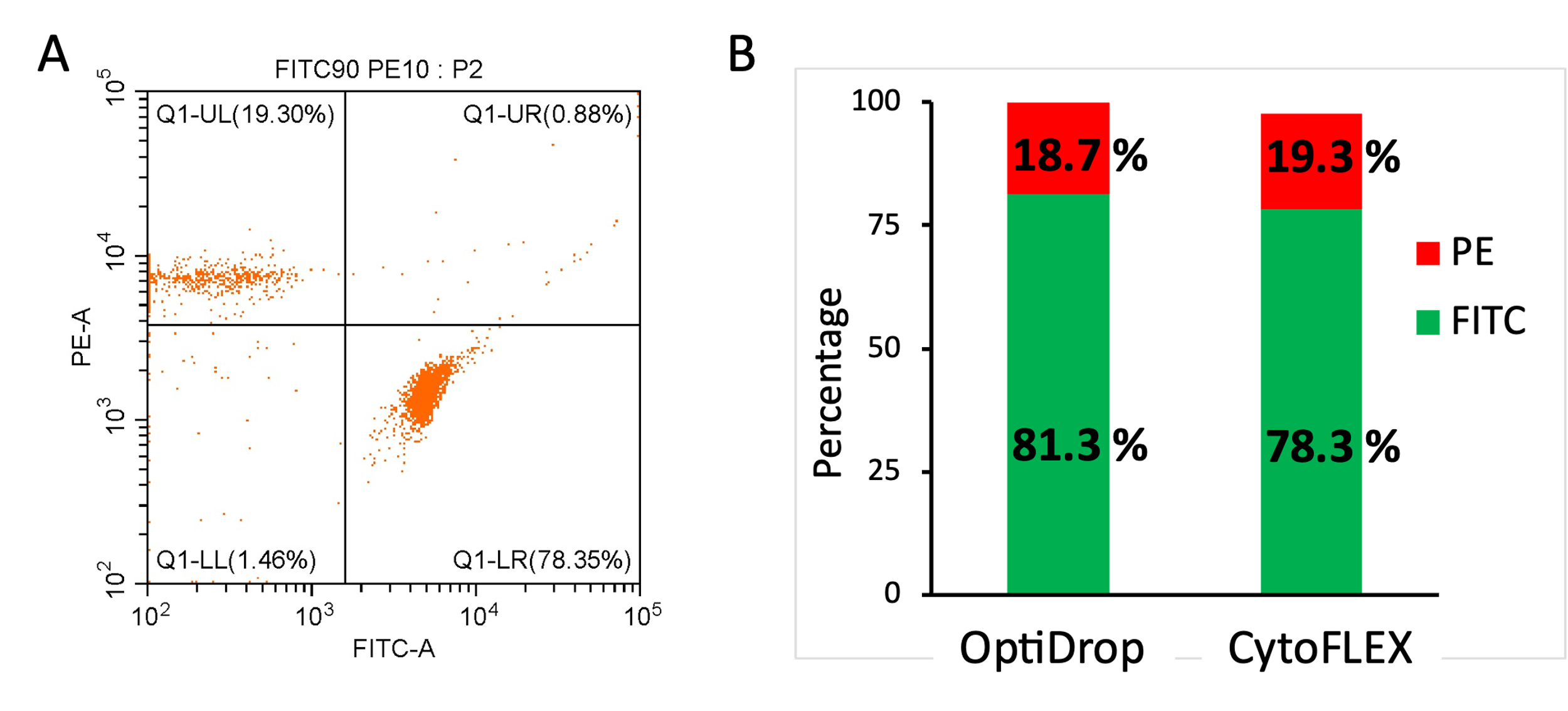


Figure S4: Comparison of OptiDrop data on mixed beads with that acquired on the CytoFLEX standard flow cytometer


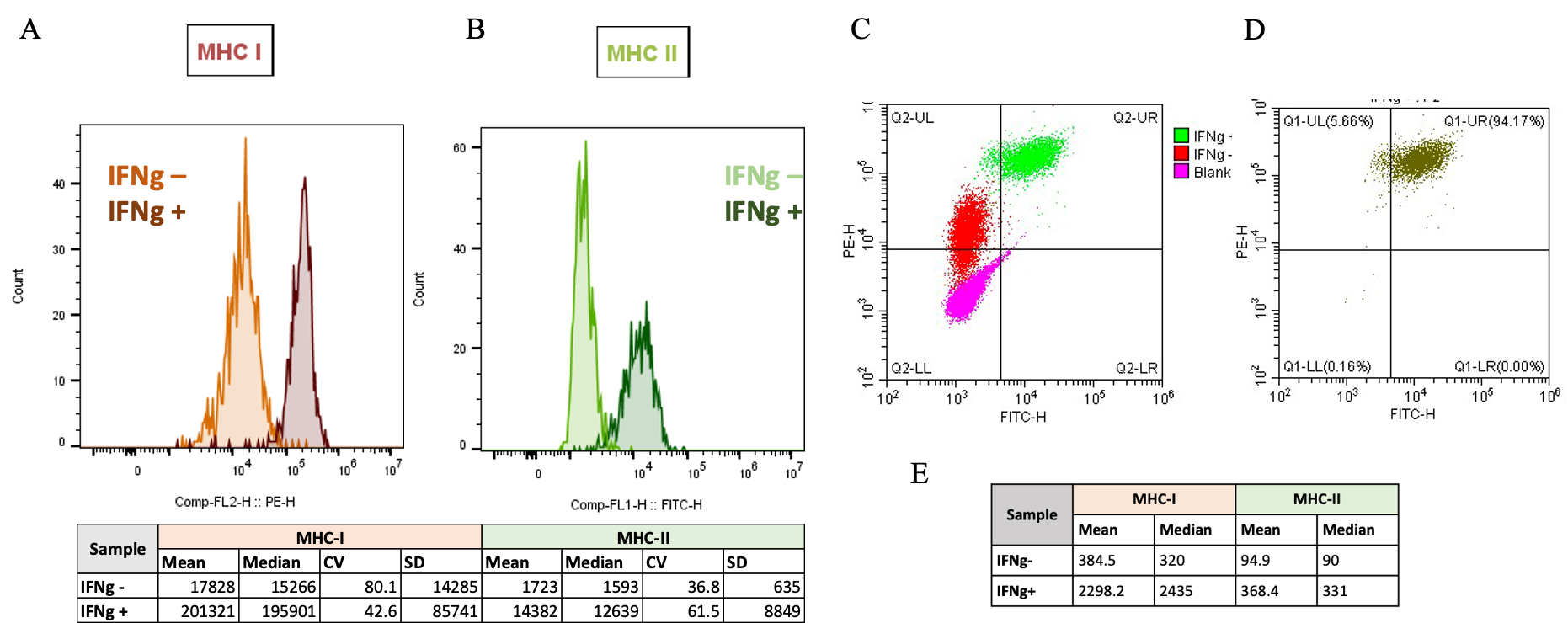


Figure S5: MEF MHC staining data obtained on standard flow cytometer (Cytoflex) (panels A - D) is comparable to that obtained on OptiDrop platform (E). A) and B) Histogram of MHC I and MHC II staining on Cytofelx along with data statistics. C) Scatter plot of unstained blank, and MHC I, MHC II stained unstimulated (IFNg-) and stimulated (IFNg+) MEFs. D) Scatter plot indicating 94% of the stimulated cells are both FITC and PE positive. E) Data statistics for the same sample set obtained on OptiDrop.

Table S1: Comparison of OptiDrop capabilities with previously reported droplet optical analysis studies


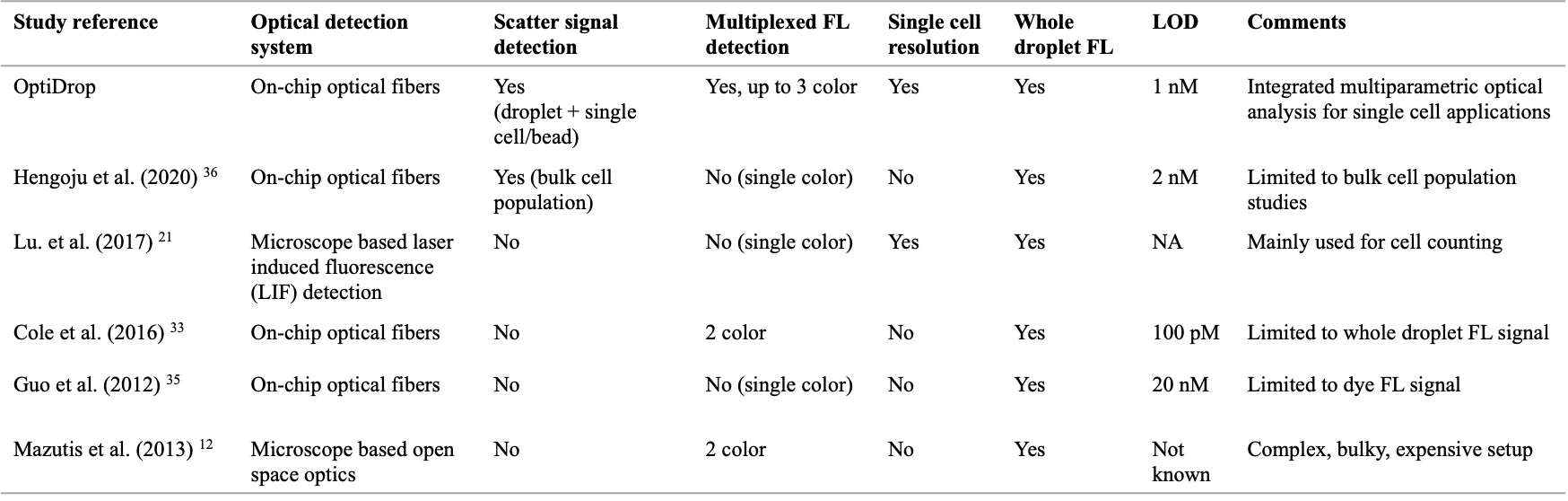

Supplement: Supplementary file 1 — Supplementary data [file 41378_2024_665_MOESM1_ESM.docx]
